# Efficacy of Immediate Lymphatic Reconstruction in Prevention of Breast Cancer‐Related Lymphedema: A Systematic Review and Meta‐Analysis

**DOI:** 10.1002/micr.70109

**Published:** 2025-08-27

**Authors:** May X. Li, Jason Zhang, Michael A. Howard, Chad M. Teven

**Affiliations:** ^1^ Feinberg School of Medicine Northwestern University Chicago Illinois USA; ^2^ Division of Plastic Surgery, Department of Surgery Northwestern Medicine Lake Forest Hospital Lake Forest Illinois USA

**Keywords:** immediate lymphatic reconstruction, LYMPHA, lymphedema

## Abstract

**Background:**

Immediate lymphatic reconstruction (ILR) is a technique in which lymphatics are visualized and lymphovenous bypass is done at the time of axillary lymph node dissection (ALND) to prevent breast cancer‐related lymphedema (BCRL). This meta‐analysis estimates the benefit of ILR in preventing lymphedema by incorporating double‐ and single‐arm studies and stratifying by length of follow‐up time.

**Methods:**

Three databases were queried for studies with primary data on ILR. Both double‐ and single‐armed studies were included, and papers with small sample sizes, overlapping samples, and unreported data were excluded. Treatment effects were calculated with risk ratios and converted to a logarithmic scale. A meta‐analysis was performed using the inverse variance method and a random‐effects model, with further analysis done by study design and length of follow‐up time.

**Results:**

A total of 17 studies were included (9 double‐arm and 8 single‐arm; *n* = 2607). The pooled treatment effect of ILR, expressed as log risk ratio (95% CI), was −0.89 (−1.18, −0.60; *p* < 0.0001). This corresponds to a relative risk of 0.41 (0.31, 0.55) and a number needed to treat of 9. Double‐ and single‐arm studies showed no significant differences in effect sizes. Studies with < 1‐year follow‐up demonstrated a larger effect size than those with longer follow‐up, and the benefits of ILR were no longer significant past 3 years.

**Conclusion:**

Patients receiving ILR were significantly less likely to develop BCRL than those receiving ALND alone. Further work is needed to examine whether benefits can truly be sustained long‐term.

## 1 Introduction

Axillary lymph node dissection (ALND) and adjuvant radiation therapy are major risk factors for postoperative upper extremity breast cancer‐related lymphedema (BCRL), a condition characterized by chronic inflammation and swelling of tissue due to disrupted lymphatic circulation (Grada and Phillips 2017; Ribeiro Pereira et al. 2017). Estimates of incidence vary, but one meta‐analysis found that 20% of patients developed unilateral arm lymphedema after ALND (DiSipio et al. 2013). These patients experience increased skin tension and volume, uncomfortable heaviness, and a diminished quality of life.

There is no cure for lymphedema, but treatment strategies such as manual lymphatic drainage, compression therapy, and exercise are widely used for conservative management (Thompson et al. 2021). Surgical techniques such as lymphaticovenular anastomosis (LVA) and vascularized lymph node transfer (VLNT) that re‐establish lymphatic circulation have also been utilized as therapy (de Sire et al. 2022). These treatment regimens are traditionally implemented post‐surgery following the development of lymphedema; however, recent years have begun shifting toward prevention. Immediate lymphatic reconstruction (ILR) utilizes LVA during breast surgery and ALND to prevent postoperative lymphedema (Boccardo et al. 2009; Johnson and Singhal 2018; Coriddi et al. 2021). First described by Boccardo et al. (2009) and since expanded upon, ILR has demonstrated promising early results.

Early studies on ILR have shown encouraging results in preventing lymphedema development, but the efficacy of the technique has not been fully elucidated. The aim of this meta‐analysis is to examine the benefit of ILR in preventing BCRL by incorporating the most recent work, including both single‐ and double‐arm studies, and evaluating the effects of follow‐up time.

## 2 Methods

### 2.1 Search Strategy

PubMed, Embase, and Scopus were searched using the terms “lymphedema” and “prevention” and “surgery” and “breast cancer” for studies with primary ILR data. Our primary outcome was the incidence of lymphedema development. Both double‐arm (comparing ILR to ALND only) and single‐arm (ILR only) studies were included. Exclusion criteria included non‐ILR interventions, non‐English papers, nonhuman subjects, no primary data, small sample size (< 20 patients), reviews, and abstracts.

A total of 1680 records were identified, 1050 were screened, 63 underwent full‐text review, and 15 were included from the database search. Ten did not report lymphedema incidence, four were excluded due to small sample size, and seven were excluded due to overlapping samples. Studies sharing common authors, originating from the same institution, with overlapping dates of data collection were deemed to have overlapping samples. In these cases, only the most recent paper was included.

**FIGURE 1:**
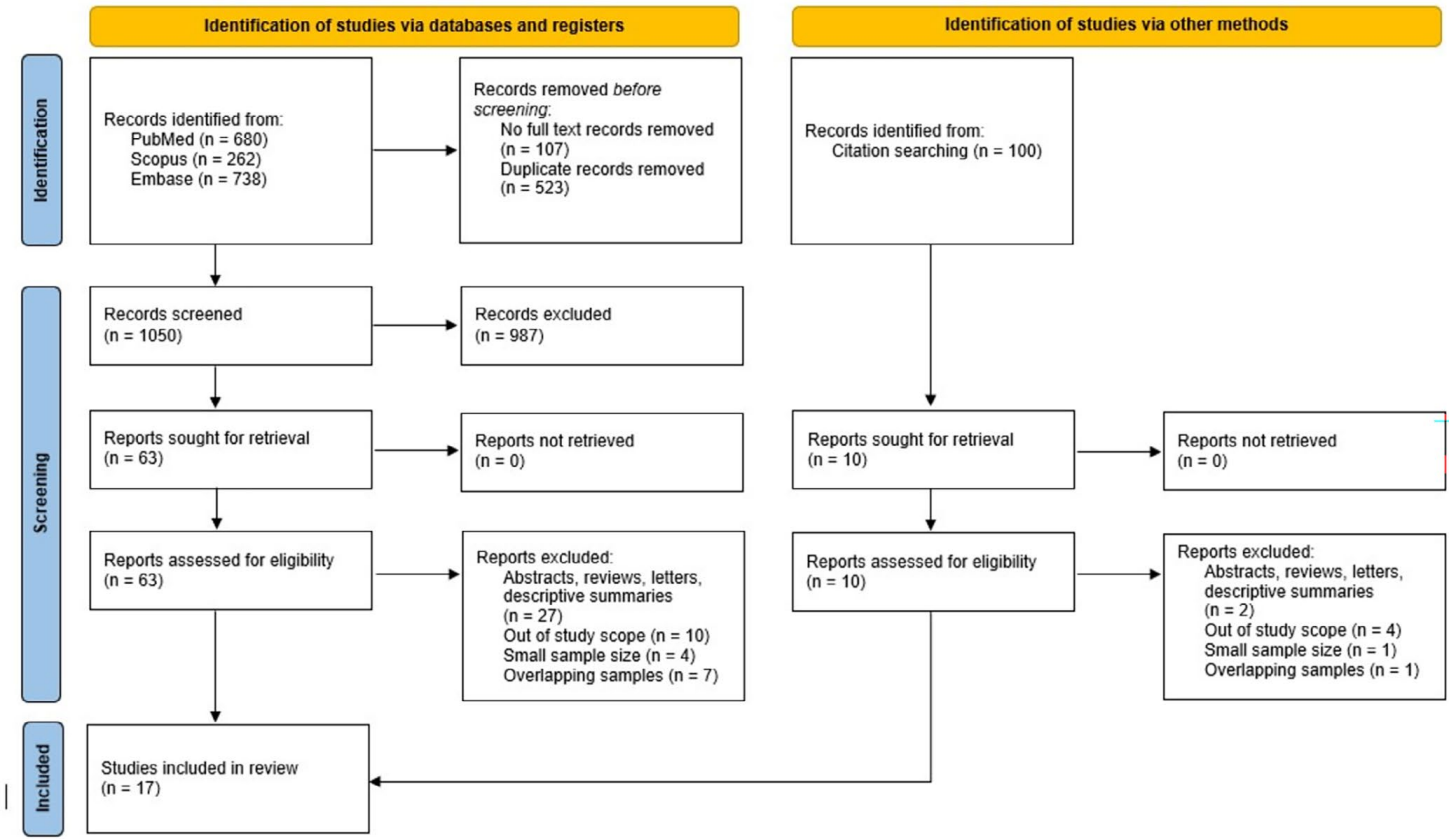
PRISMA flow diagram. *Source:* Page, M. J., J. E. McKenzie, P. M. Bossuyt, et al. 2021. “The PRISMA 2020 Statement: an Updated Guideline for Reporting Systematic Reviews.” *BMJ* 372: N71. https://doi.org/10.1136/bmj.n71.

A citation search was also conducted in PubMed for papers citing Boccardo et al. (2009) and Boccardo et al. (2011). A total of 100 records were identified and 10 were retrieved for full‐text review. Four were excluded due to unreported lymphedema incidence, one due to small sample size, and one due to overlapping patient samples. Two articles were included from the citation search, yielding a total of 17 articles included in the meta‐analysis (Boccardo et al. 2011, 2014; Haravu et al. 2024; Le et al. 2024; Coriddi et al. 2023; Ozmen et al. 2022; Herremans et al. 2021; Chung et al. 2023; Levy et al. 2023; Wong et al. 2024; Schwarz et al. 2019; Shaffer et al. 2020; Spoer et al. 2024; Granoff et al. 2023; Cook et al. 2021; Wainwright et al. 2024; Brahma et al. 2024) (Figure 1).

### 2.2 Risk of Bias

A risk of bias assessment was conducted using the Cochrane RoB 2.0 tool (Sterne et al. 2019) for randomized controlled studies and the ROBINS‐I tool (Sterne et al. 2016) for nonrandomized interventions. The Cochrane RoB 2.0 tool assessed bias in five domains (randomization process, deviations from intended intervention, missing outcome data, measurement of outcome, and selection of reported results). The ROBINS‐I tool assessed bias in seven domains (confounding, selection of participants, classification of interventions, deviation from intended interventions, missing data, measurement of outcomes, and selection of reported results).

### 2.3 Statistical Methods

Effect sizes for lymphedema development in the ILR versus ALND‐only group were calculated using risk ratios from the given sample data and converted to a logarithmic scale to normalize the distribution of values for analysis. Data from Coriddi et al. (2023) were preliminary results from a prospective trial reported with Kaplan–Meier curves; thus, incidence was calculated using data from their longest follow‐up. To calculate treatment effects in single‐arm studies, the incidence of BCRL following ALND only was taken from the literature (DiSipio et al. 2013).

A meta‐analysis was performed in R (meta package version 8.0‐1) (Balduzzi et al. 2019) using the inverse variance method and a random‐effects model. Treatment effects were reported as log risk ratios with 95% confidence intervals (CIs). Separate subgroup analyses were performed based on whether the study was a double‐arm or a single‐arm study. Papers were further categorized by follow‐up time, and subgroup analyses compared the effect sizes of samples with less than 1 year of follow‐up and greater than 2‐ and 3‐year follow‐up. Follow‐up length was determined based on the reported average or median follow‐up time. Wainwright et al. (2024) did not report follow‐up time and was not included in this analysis. A funnel plot was generated to assess for publication bias.

## 3 Results

### 3.1 Study Characteristics

A total of 17 studies (Boccardo et al. 2011, 2014; Haravu et al. 2024; Le et al. 2024; Coriddi et al. 2023; Ozmen et al. 2022; Herremans et al. 2021; Chung et al. 2023; Levy et al. 2023; Wong et al. 2024; Schwarz et al. 2019; Shaffer et al. 2020; Spoer et al. 2024; Granoff et al. 2023; Cook et al. 2021; Wainwright et al. 2024; Brahma et al. 2024) (*n* = 2607) were included. Nine studies were double‐arm (*n* = 468 ILR; *n* = 1373 non‐ILR) and eight were single‐arm (*n* = 766). Two were randomized controlled trials, four were prospective observational studies, and 11 were retrospective studies. Twelve studies were conducted in the US, two in Italy, and one in Korea, Singapore, and Indonesia (Tables 1 and 2).

**TABLE 1:** Table of study characteristics for double‐arm studies (Boccardo et al. 2011; Haravu et al. 2024; Le et al. 2024; Coriddi et al. 2023; Ozmen et al. 2022; Herremans et al. 2021; Chung et al. 2023; Levy et al. 2023; Wong et al. 2024).

Study	Country	Design	Study period	ILR	Control	Participant demographics	Oncologic therapy	Operation time	No. bypasses	No. nodes removed	Follow‐up time	LE incidence	Measure	Other complications
Boccardo et al. (2011)	Italy	RCT	Mar 2008–Sep 2009	*n* = 23 intussusception	*n* = 23	Age, median (IQR) 68 (52–74) ILR 67 (53–73) control BMI, median (IQR) 29 (24–32) ILR 26 (23–32) control	Radiotherapy 47.8% ILR 52.2% control Chemotherapy NR	LVB 15–20 min Overall NR	2–4	Median (IQR) 17 (14–24) ILR 18 (12–22) control	18 months	4.3% ILR 30.4% control	Vol LS	Cellulitis 17.4% ILR 21.7% control
Haravu et al. (2024)	USA	Retro	Jul 2019–Sep 2021	*n* = 69 intussusception	*n* = 782	Age, mean (SD) 57.7 (13.5) ILR 59.9 (13.6) control BMI, mean (SD) 28.4 (7.4) ILR 27.5 (6.6) control	Radiotherapy 87.6% ILR NR control Chemotherapy 92.1% ILR NR control	NR	Mean (SD) 1.1 (0.3)	Mean (SD) 15.4 (5.9) ILR NR control	Mean 311 days	10.9% ILR 50.1% Control	Vol LC	NR
Le et al. (2024)	USA	Pro	2016–2019	*n* = 77 intussusception	*n* = 94	Age, mean (SD) 53 (12) ILR 51 (11) control BMI, mean (SD) 27.2 (5.3) ILR 28.6 (5.3) control	Radiotherapy 90.9% ILR 61.7% control Chemotherapy 93.5% ILR 100% control	LVB NR Overall, mean (SD), min 226.0 (11.8) ILR 126.8 (38.8) control	Mean (SD) 2.90 (1.14)	NR	24 months	10.4% ILR 38.3% Control	LC BIS	Infection 1.3% ILR 5.3% control
Coriddi et al. (2023)	USA	RCT	Jan 2020–Mar 2023	*n* = 50 intussusception	*n* = 49	Age, mean (SD) 48.5 (11.3) ILR 46.3 (11.4) control BMI, mean (SD) 27.5 (5.7) ILR 27.1 (4.9) control	Radiotherapy 90.0% ILR 91.8% control Chemotherapy 84.0% ILR 89.8% control	NR	Mean (SD) 2.7 (NR)	Mean (SD) 18.6 (6.7) ILR 18.4 (7.0) control	24 months	19.0% ILR 68.4% control	Vol BIS ICGL	NR
Ozmen et al. (2022)	USA	Retro	Jan 2014–Nov 2020	*n* = 110 SLYMPHA	*n* = 84	Age, mean (SD) 50 (12) ILR 53 (10) control BMI, mean (SD) NR	Radiotherapy 80.1% ILR 80.1% Control Chemotherapy NR	NR	NR	Mean (SD) 17.57 (7) ILR 18.36 (8) control	Mean (SD), months 29 (18) ILR 70 (44) control	16.4% ILR 32.1% control	BIS	NR
Herremans et al. (2021)	USA	Retro	2014–2021	*n* = 76 intussusception	*n* = 56	Age, mean (SD) 55.2 (13.9) ILR 58.2 (10.1) control BMI, mean (SD) 27.8 (6.3) ILR 29.4 (7.5) control	Radiotherapy 88.2% ILR 89.3% control Chemotherapy 76.3% ILR 75.0% control	NR	NR	Mean (SD) 17.4 (6.5) ILR 18.5 (8.8) control	Mean (SD), months 30.3 (13.9) ILR 55.3 (18.8) control	13.2% ILR 28.6% control	LC BIS	NR
Chung et al. (2023)	Korea	Retro	Nov 2019–Feb 2021	*n* = 26 intussusception, end‐to‐side	*n* = 183	Age, mean (SD) 52.27 (10.0) ILR 52.10 (12.0) control BMI, mean (SD) 24.57 (3.06) ILR 23.44 (3.69) control	Radiotherapy 60.0% ILR 22.4% control Chemotherapy 93.3% ILR 56.3% control	NR	1	NR	Mean 14 months	3.8% ILR 7.7% control	LC LS	NR
Levy et al. (2023)	USA	Retro	Nov 2012–Nov 2016	*n* = 45 intussusception	*n* = 45	Age, mean (SD) 54.2 (11.0) ILR 51.2 (12.7) control BMI, mean (SD) 27.7 (6.8) ILR 29.9 (7.3) control	Radiotherapy 60.0% ILR 68.9% control Chemotherapy 100.0% ILR 97.8% control	NR	Mean (SD) 1.6 (0.8)	Mean (SD) 15.8 (6.8) ILR 14.4 (6.4) control	Median (IQR), months 57.0 (31.0–74.5) ILR 63.0 (47.5–80.0) control	31.1% ILR 33.3% control	LC BIS	NR
Wong et al. (2024)	Singapore	Retro	Jan 2018–Dec 2021	*n* = 26 dLYMPHA	*n* = 87	Age, mean (SD) 54 (11.2) ILR 59 (13.0) control BMI, mean (SD) 25.2 (4.12) ILR 24.0 (4.82) control	Radiotherapy 73.1% ILR 65.5% control Chemotherapy 84.6% ILR 50.6% control	LVB NR Overall, mean (SD), min 120 (NR) ILR NR control	Mean (range) 3 (1–5)	Mean (range) 18.2 (1–38) ILR 14.2 (1–47) control	Mean 38.5 months	3.8% ILR 17.2% control	LC LS	NR

Abbreviations: BIS, bioimpedance spectroscopy; ICGL, ICG lymphography; LC, limb circumference; LS, lymphoscintigraphy; NR, not reported; Vol, volumetry.

**TABLE 2:** Table of study characteristics for single‐arm studies (Schwarz et al. 2019; Boccardo et al. 2014; Shaffer et al. 2020; Spoer et al. 2024; Granoff et al. 2023; Cook et al. (2021); Wainwright et al. 2024; Brahma et al. 2024).

Study	Country	Design	Study period	ILR	Participant demographics	Oncologic therapy	Operation time	No. bypasses	No. nodes removed	Follow‐up time	LE incidence	Measure	Other complications
Schwarz et al. (2019)	USA	Retro	Sep 2016–Dec 2018	*n* = 58 end‐to‐end, intussusception	Age, mean (range) 51.7 (31–78) BMI, mean (SD) NR	Radiotherapy 89% Chemotherapy 74%	LVB, mean (range), min 85 (40–150) Overall NR	Mean (range) 1.4 (1–4)	Mean (range) 14 (5–41)	Median (range), months 11.8 (1–29)	3.4%	LC BIS	Dermatitis 1.7% Infection 1.7%
Boccardo et al. (2014)	Italy	Pro	Jul 2008–Dec 2012	*n* = 74 intussusception	Age, median (IQR) 57 (42–69) BMI, median (IQR) 24 (21–33)	Radiotherapy 47.3% Chemotherapy NR	LVB, mean (SD), min 20 (NR) Overall NR	2–4	Median (IQR) 19 (12–21)	4 years	4.1%	Vol LS	Cellulitis 18.9%
Shaffer et al. (2020)	USA	Pro	Sep 2016–Nov 2019	*n* = 88 end‐to‐end, intussusception	Age, mean (SD) 51 (11.4) BMI, mean (SD) 27.8 (5.9)	Radiotherapy 93% Chemotherapy 93%	LVB, mean, min 212 in 2016 87 in 2019 Overall, mean, min 212 in 2016 160 in 2019	Mean (SD) 2.15 (1.16)	Mean (SD) 14.5 (6.67)	Mean (SD), months 14.6 (NR)	5.7%	LC BIS	NR
Spoer et al. (2024)	USA	Retro	Jan 2018–Aug 2022	*n* = 32 end‐to‐end, intussusception, end‐to‐side, double‐barrel, coupler‐assisted	Age, mean (SD) 54.4 (11.8) BMI, median (IQR) 30.2 (27.1–34.9)	Radiotherapy 84.4% Chemotherapy 96.9%	LVB NR Overall, mean (SD), min 333.8 (84.1)	Median (IQR) 2 (1–2.5)	Median (IQR) 15 (11–21)	Median (IQR), months 8.1 (6.9–11.5)	6.3%	LC BIS	Seroma 6.3% Delayed healing 3.1% Dehiscence 6.3% Infection or cellulitis 18.8% Reoperation 15.6% Cancer recurrence 3.1% Mortality 12.5%
Granoff et al. (2023)	USA	Retro	Sep 2016–Sep 2020	*n* = 90 intussusception	Age, mean (SD) 53.9 (12.5) BMI, median (IQR) 26.6 (24.0–29.9)	Radiotherapy 86.7% Chemotherapy 66.7%	NR	Median (IQR) 1 (1–2)	Median (IQR) 14 (8–19)	Median (range), months 17 (6–49)	8.9%	Vol LC BIS	NR
Cook et al. (2021)	USA	Retro	2017–2019	*n* = 33 intussusception	Age, mean (SD) NR BMI, mean (SD) 29.6 (5.2)	Radiotherapy 66.7% Chemotherapy 72.7%	NR	Mean (SD) 1.7 (0.9)	Mean (SD) 15.2 (6.3)	Mean (range), days 299.2 (9–760)	9.1%	LC	Cellulitis 0.0%
Wainwright et al. (2024)	USA	Pro	May 2018–May 2023	*n* = 341 intussusception	Age, mean (SD) 52 (12) BMI, mean (SD) NR	Radiotherapy 88.9% Chemotherapy 94.4%	LVB NR Overall, mean (SD), min 222.3 (56.9)	Mean (SD) 3.1 (1.4)	NR	NR	9.4%	LC BIS	Seroma 5.0% Hematoma 1.5% Infection 3.2%
Brahma et al. (2024)	Indonesia	Retro	May 2020–Feb 2023	*n* = 50 end‐to‐end, intussusception, end‐to‐side, buffalo skull‐shape, Y‐shape, lymphaticolymphatic	Age, mean (SD) 50 (12) BMI, mean (SD) 25.8 (4.6)	Radiotherapy 70.7% Chemotherapy 91.5%	LVB, median (range), min 60 (17–290) Overall, median (range), min 270 (145–705)	Mean (SD) 1.68 (0.9)	Median (range) 13 (6–31)	Median (range), months 12.5 (1–33)	22.0%	ICGL	NR

Abbreviations: BIS, bioimpedance spectroscopy; ICGL, ICG lymphography; LC, limb circumference; LS, lymphoscintigraphy; NR, not reported; Vol, volumetry.

The average number of bypasses performed was 2.32, and the average number of lymph nodes removed was 15.64 (pooled from 11 and nine studies with adequate reporting, respectively). Most studies utilized an intussusception technique (also described as a sleeve, telescoping, dunking, or parachuting) in which one or more dissected lymphatic vessels were gathered into the cut end of a vein. A few studies performed true intima‐to‐intima anastomoses if a precise size match existed. Brahma et al. (2024) also utilized lymphaticolymphatic anastomosis to efferent lymphatics, and Spoer et al. (2024) compared results of a coupler‐assisted bypass to the “standard” LVB technique. Wong et al. (2024) employed a distally‐based technique (dLYMPHA) utilizing venules in the distal upper extremity for easier vessel access, and Ozmen et al. (2022) performed a simplified version of LYMPHA (SLYMPHA) that did not require an operating microscope.

### 3.2 Risk of Bias

**FIGURE 2:**
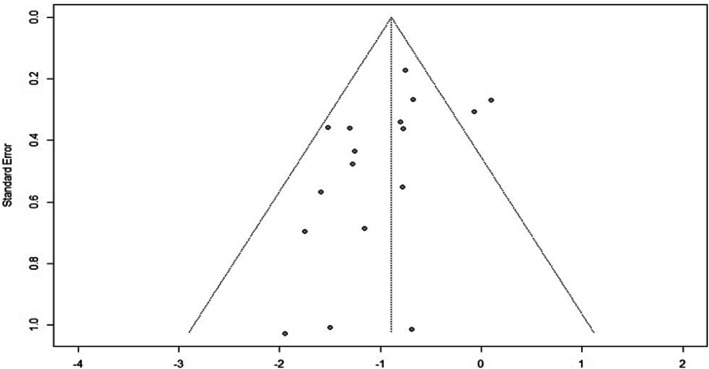
Funnel plot to assess for publication bias.

Of the two RCTs evaluated, Coriddi et al. (2023) demonstrated low overall risk of bias, while Boccardo et al. (2011) demonstrated moderate risk due to an incomplete description of their randomization process. Of the 15 nonrandomized interventions, 9 studies had a moderate overall risk and 6 studies had a low risk. No studies demonstrated a serious overall risk of bias, although four studies did have a serious risk in one of the seven evaluated domains (Tables S1 and S2). A funnel plot demonstrated slight asymmetry, indicating possible publication bias (Figure 2).

### 3.3 Effect of ILR on Lymphedema Incidence

**FIGURE 3:**
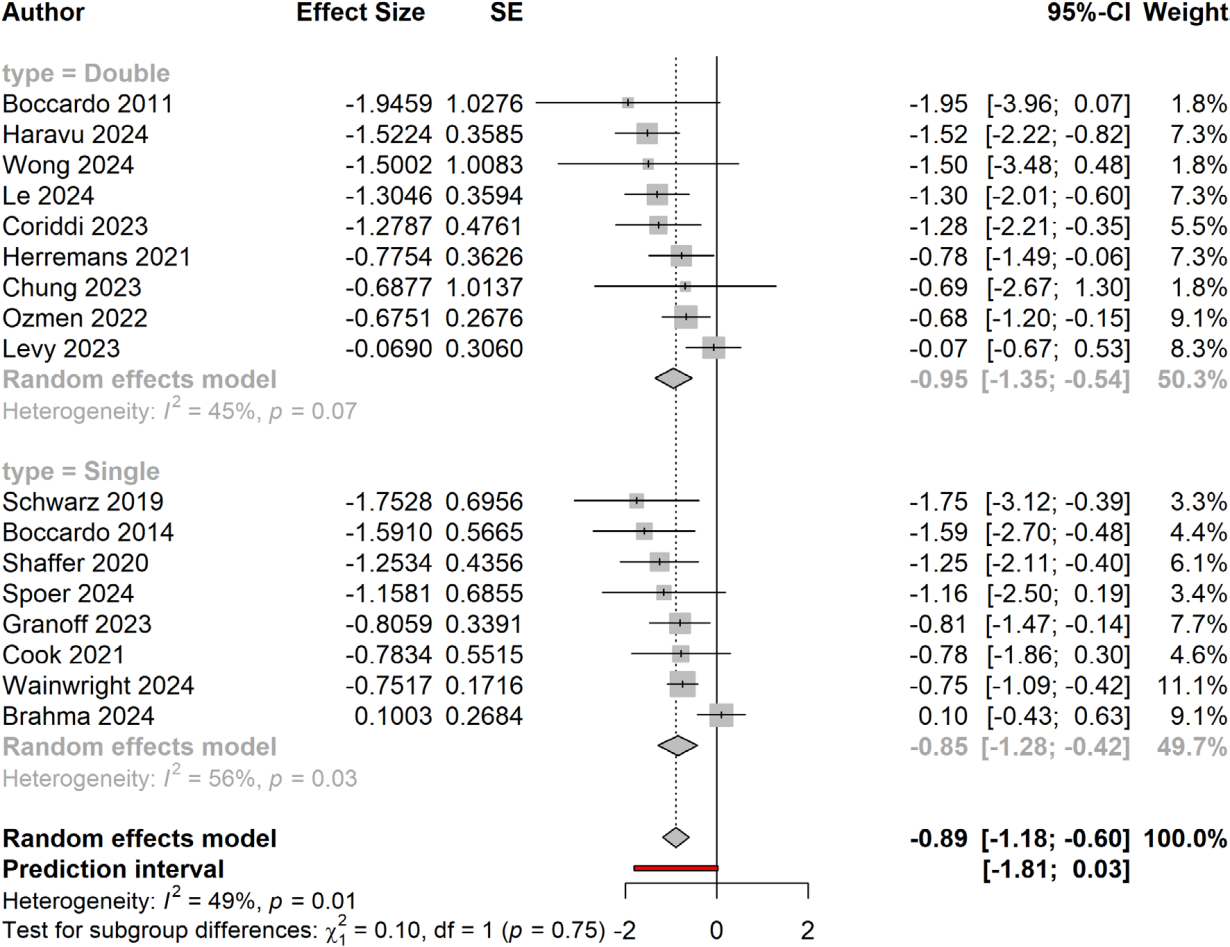
Forest plot showing treatment effect of ILR, separated by double‐arm studies (top) and single‐arm studies (bottom).

Meta‐analysis demonstrates a significant pooled effect size of −0.89 (95% CI: −1.18, −0.60; *p* < 0.0001), which corresponds to a relative risk (RR) of 0.41 (0.31, 0.55). Double‐ and single‐arm studies contributed equally in weight (50.3% and 49.7%, respectively). When separated by study design, double‐arm studies alone demonstrated a greater treatment effect of −0.95 (−1.35, −0.54; *p* < 0.0001), corresponding to an RR of 0.39 (0.26, 0.58). Single‐arm studies alone had an effect size of −0.85 (−1.28, −0.42; *p* = 0.0001), corresponding to an RR of 0.43 (0.28, 0.66). Subgroup analysis demonstrated no significant difference between the effect sizes of double‐ and single‐arm studies (*p* = 0.75). Overall and regardless of study design, ILR demonstrated significantly lower rates of lymphedema, with those undergoing ILR 0.41 times as likely to develop BCRL compared to those with ALND alone (Figure 3).

### 3.4 Effect of Follow‐Up Time

**FIGURE 4:**
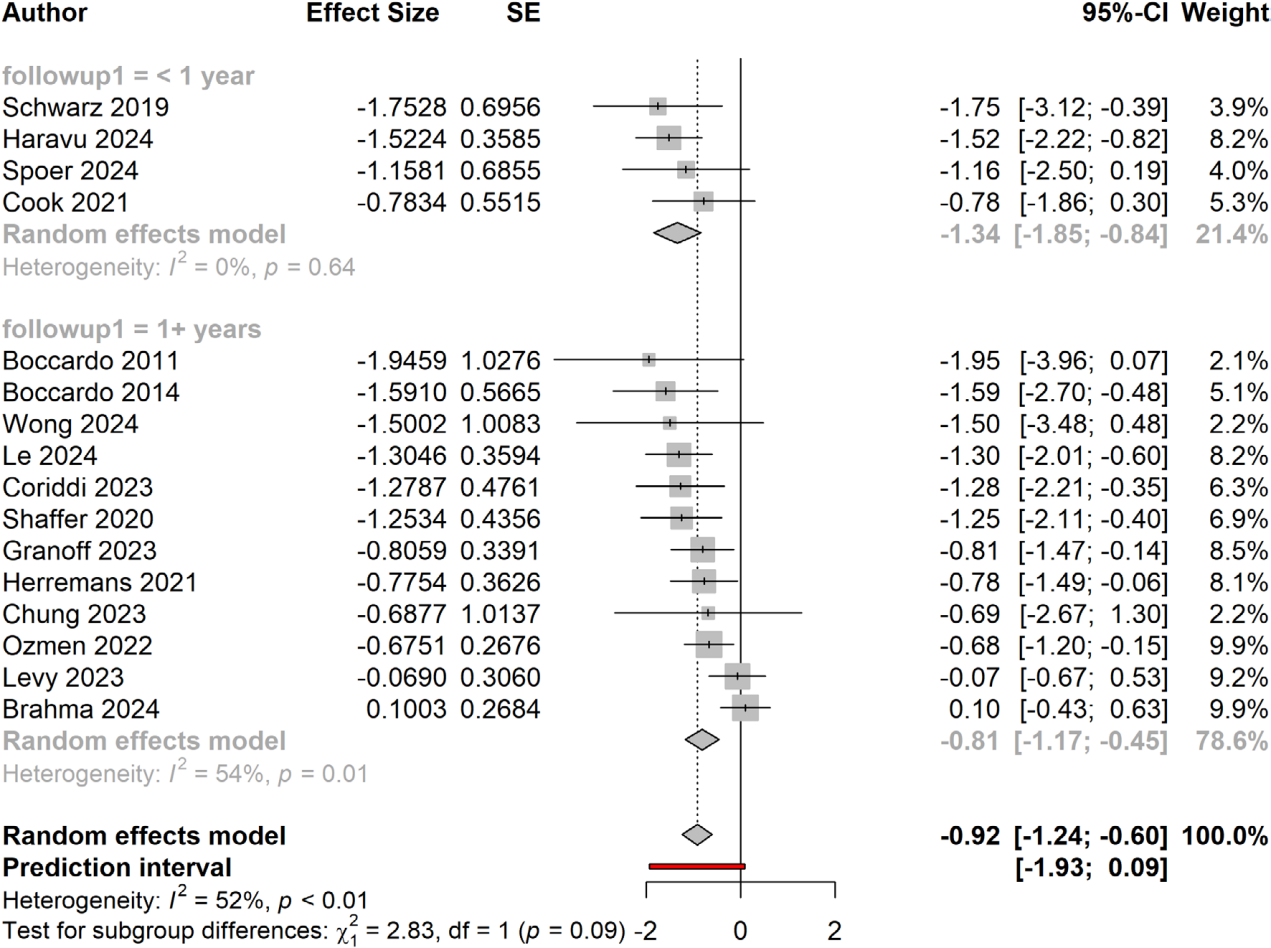
Forest plot showing the effect size of ILR in studies with less than 1‐year follow‐up (top) versus studies with at least 1‐year follow‐up (bottom).

Studies with less than 1‐year follow‐up demonstrated a larger effect size than those with more than 1‐year follow‐up (effect size of −1.34, equal to RR of 0.26 vs. effect size of −0.81, equal to RR of 0.44, respectively; Figure 4). A test for subgroup differences showed that this approached significance (*p* = 0.09). Excluding papers with fewer than 1‐year follow‐up yielded an effect size of −0.81 (−1.17, −0.45), which is notably not significantly different from the results of our overall meta‐analysis.

**FIGURE 5:**
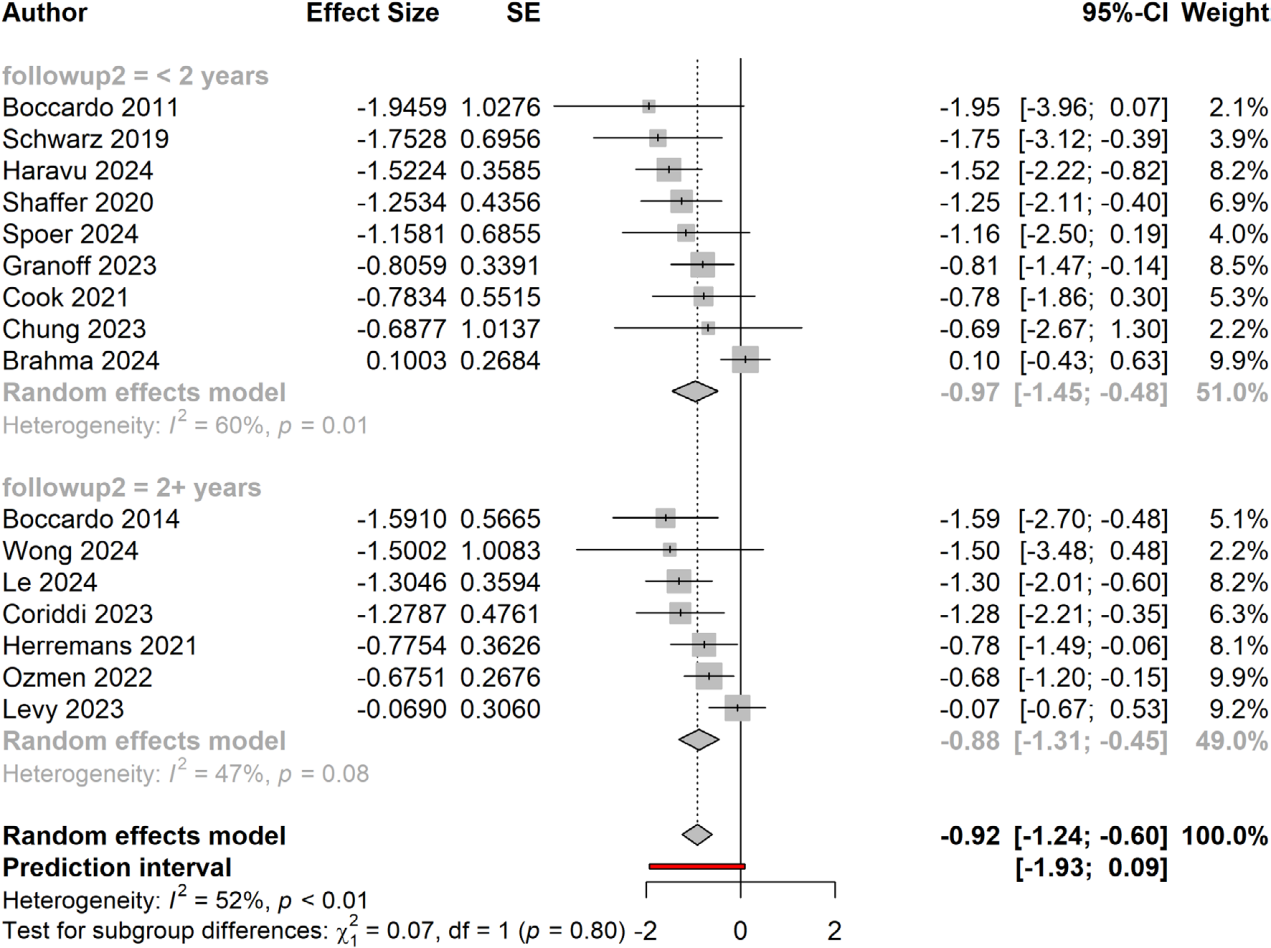
Forest plot showing the effect size of ILR in studies with less than 2‐year follow‐up (top) versus studies with at least 2‐year follow‐up (bottom).

**FIGURE 6:**
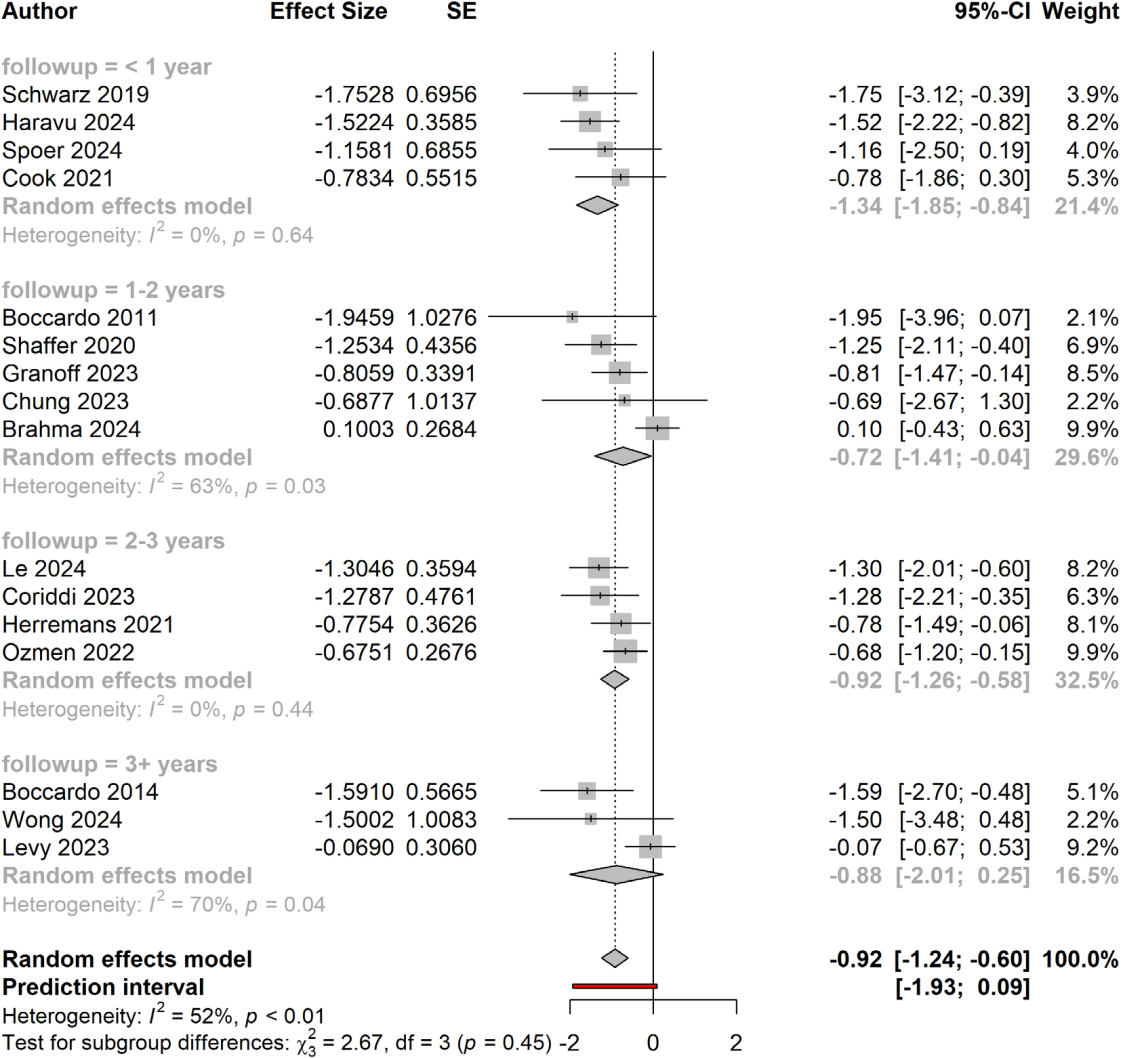
Forest plot comparing effect sizes of studies broken down by length of follow‐up time by yearly intervals.

Studies with less than 2‐year follow‐up still demonstrated a larger effect size than those with greater than 2‐year follow‐up; however, the difference between groups was not significant (effect size of −0.97, equal to RR of 0.38 vs. effect size of −0.88, equal to RR of 0.41; *p* = 0.80; Figure 5). Figure 6 shows the effect sizes of studies by follow‐up time broken down into yearly intervals. Three papers had follow‐up times of at least 3 years, and they demonstrated a pooled effect size of −0.88 (−2.01, 0.25). Notably, while this value stayed consistent, the effect size past 3 years was no longer statistically significant (*p* = 0.126).

## 4 Discussion

### 4.1 Length of Follow‐Up

Our meta‐analysis demonstrates an overall 59% RR reduction in BCRL among patients undergoing ILR compared to ALND alone. This corresponds to a number needed to treat of 9, based on calculations with the estimated 20% incidence rate of BCRL reported in the literature. While this finding is consistent with previous meta‐analyses on ILR (Chun et al. 2022; Hill et al. 2022; Wong et al. 2025), ours is the first to analyze studies by length of follow‐up.

Upon subgroup comparison by follow‐up time, studies reporting less than 1‐year follow‐up demonstrated larger effect sizes than studies with longer follow‐up at a rate approaching significance (*p* < 0.1; Figure 4). Exclusion of studies with less than 1‐year follow‐up, however, did not change the overall results of our meta‐analysis. The benefit of ILR continued to remain significant among papers with at least 2 years of follow‐up (effect size of −0.88; *p* < 0.0001; Figure 5). Notably, three studies with at least 3‐year follow‐up continued to demonstrate the same effect size; however, this effect was no longer statistically significant (*p* = 0.126; Figure 6).

Overall, these findings suggest that the benefits of ILR are greater in the short term, but there may be no significant benefit to ILR in the long term. ILR demonstrated significant benefit in reducing BCRL in a pooled analysis of all included studies; however, when studies with at least 3 years of follow‐up were analyzed separately, the effects of ILR were no longer significant. As only three studies had at least 3 years of follow‐up, more studies with longer follow‐up are needed to determine whether the effects of ILR can truly be sustained over time. Individual studies also report mixed results on ILR efficacy and time to lymphedema development. While we were unable to incorporate their results into our meta‐analysis, Jakub et al. (2024) conducted a prospective quasi‐randomized trial and found no benefit to ILR in decreasing BCRL. In a direct comparison of their results to the randomized trial from Coriddi et al. (2023), they describe markedly different results at the same time points using the same diagnostic criteria. Specifically, Coriddi et al. (2023) reported a cumulative lymphedema incidence of 2% at 12 months and 9.5% at 24 months, while Jakub et al. (2024) had rates of 17% and 36% at 12 and 24 months, respectively.

Herremans et al. (2021) directly compared time to lymphedema development between ILR and non‐ILR patients within their own study. They found that the majority (73%) of patients who developed lymphedema did so within the first year, and there were no significant differences in time to lymphedema diagnosis between ILR and non‐ILR patients. However, a larger proportion of ILR patients experiencing BCRL were diagnosed at later time points. Specifically, 75% of non‐ILR patients were diagnosed at 0–11 months compared to 70% of ILR patients. At 12–35 and 36–60 months, 18.8% and 6.2% of non‐ILR patients were diagnosed compared to 20% and 10% of ILR patients, respectively. Thus, among patients who experienced BCRL, more ILR patients tended to be diagnosed later than non‐ILR patients. Though these variations were based on limited sample sizes and not statistically significant, this delayed presentation of lymphedema following ILR may warrant further research.

Overall, there are considerable variations between studies and a paucity of long‐term work that makes it difficult to conclude whether ILR truly prevents or only delays progression to lymphedema. Even so, the short‐term benefit of ILR has clearly been demonstrated, and an effective delay in lymphedema progression still benefits patients by reducing cancer‐related morbidity, especially when the majority of those experiencing BCRL are diagnosed within the first year following ALND.

### 4.2 Lymphedema Risk Factors

While ALND, regional radiation, and taxane‐based chemotherapy are known risk factors for BCRL, there are several individualized factors, such as subclinical edema, cellulitis, and BMI, that increase the risk for lymphedema (Gillespie et al. 2018). Among our included studies, all patients underwent ALND, all studies reported rates of radiation, and 14 of our 17 studies reported rates of chemotherapy. Rates of subclinical edema were not included or not reported, and only four studies reported rates of cellulitis. A total of 15 studies had patient BMI, with 11 reporting mean/median patient BMI as overweight according to WHO guidelines. Patients with these risk factors are known to be at higher risk for postoperative lymphedema; however, how these risk factors may specifically modify the efficacy of ILR is less clear.

Wainwright et al. (2024) specifically examined the effect of obesity (BMI ≥ 30 kg/m^2^), identifying BMI as a risk factor that may affect the success and efficacy of ILR. They found that patients who developed BCRL after ILR were significantly more likely to have obesity than patients without BCRL (56.3% vs. 34.5%, respectively; *p* = 0.04). The authors also found significantly increased odds of ILR procedural failure in patients with obesity (OR 2.6; *p* = 0.01). Even so, it should be highlighted that patients with obesity in this study still had lower rates of BCRL after ILR than patients after ALND alone.

Other authors have found mixed results regarding the effect of BMI in ILR. Haravu et al. (2024) found that ILR was slightly more likely to be aborted in those with an elevated BMI; however, these results were not significant (*p* = 0.08). They also found no significant difference in BMI among patients who developed BCRL post‐ILR (*p* = 0.58), suggesting that BMI was not a predictor of ILR success or efficacy in their cohort. Ozmen et al. (2022) also found no significant effect of obesity among those who developed BCRL post‐ILR (25% and 23% with and without obesity, respectively; *p* = 0.79).

Herremans et al. (2021) uniquely conducted a bivariate logistic regression model to analyze ILR efficacy while controlling for other risk factors, including BMI, number of nodes removed, chemotherapy, and radiation. Their analysis found a significant effect of ILR (*p* < 0.05) independent of all risk factors *except* BMI (*p* = 0.062). Notably, they found that BMI was significantly greater in all patients who developed lymphedema than in patients who did not (*p* = 0.003). However, this difference was no longer significant in patients undergoing ILR (*p* = 0.084). Thus, elevated BMI may modify the success of ILR, but it does not seem to overrule the benefits of ILR in preventing lymphedema among patients with obesity who are already at higher risk.

In addition, most papers that analyzed the impact of treatment‐related risk factors on ILR efficacy found no significant association with lymphedema outcome. Cook et al. (2021) and Wainwright et al. (2024) found significant associations with the number of nodes removed and radiation treatment, respectively; however, Haravu et al. (2024), Ozmen et al. (2022), and Herremans et al. (2021) found no significant differences in lymphedema outcome based on chemotherapy, radiation, or number of nodes removed.

More research is needed to clarify what factors may impact ILR success to better inform clinical guidelines and patient selection criteria; however, current studies suggest that the utility of ILR in reducing post‐ALND morbidity far outweighs the impact of any risk factor. Specifically, the benefits of ILR seem to stand largely independent of radiation or chemotherapy receipt. Obesity may reduce the efficacy of ILR, but not enough to render the procedure ineffective. Rather, those with obesity would have the greatest potential for benefit with ILR, and clinical practice should emphasize the use of ILR in this population.

### 4.3 Limitations

Our calculated effect size relies on the results of the individual studies included; thus, papers that did not report the incidences and sample sizes of each group could not be included. For single‐arm studies, we used the reported rate of BCRL following ALND taken from the literature to calculate effect sizes; thus, this may not be reflective of the true incidence. Most included studies were also retrospective, so no causative conclusions can be drawn from their results. Furthermore, the studies analyzed had moderate heterogeneity (*I* ^2^ = 49%), and our included studies differed in patient characteristics and oncologic presentation. Studies also varied in the postoperative management of patients (i.e., physiotherapy, compression sleeve usage, post‐op instructions), which we were unable to control for and warrant more direct analysis in future studies.

Variations in the number and experience of operating surgeons and operative technique may also impact the outcome and development of lymphedema. There was a relative lack of consistency among papers regarding specific techniques used. Most studies used the intussusception technique; however, some also utilized true end‐to‐end intima‐to‐intima anastomoses in cases of precise vessel match or various other techniques in cases of vessel size mismatch (i.e., end‐to‐side, double‐barrel, buffalo skull). SLYMPHA (Ozmen et al. 2022) and dLYMPHA (Wong et al. 2024) were also notable modifications to the original technique; these modifications have fundamental differences in procedure that, while effective in each given study, have not been widely replicated and may reflect a bias toward simply publishing positive findings. A slight asymmetry in our funnel plot (Figure 2) may reflect this publication bias as well. Finally, we were not able to expand upon metrics such as oncologic safety and other complication rates due to a paucity in reporting.

## 5 Conclusion

ILR significantly reduces the incidence of BCRL following ALND within the first 3 years after surgery; however, the significant benefit disappears with longer follow‐up time. More studies with longer follow‐up are required to examine if these benefits can truly be sustained long‐term. ILR should be emphasized in high‐risk patients undergoing ALND, and future work should strive to clarify the impact of patient‐level and perioperative factors on ILR efficacy.

## Conflicts of Interest

The authors declare no conflicts of interest.

## Supporting information


**Data S1:** Supporting Information.

## Data Availability

Data sharing is not applicable to this article as no new data were created or analyzed in this study.
