# Supplementary material for: Efficacy of Immediate Lymphatic Reconstruction in Prevention of Breast Cancer‐Related Lymphedema: A Systematic Review and Meta‐Analysis
Source: Microsurgery. 2025 Aug 27;45(6):e70109. doi: 10.1002/micr.70109 (PMC12381909; doi:10.1002/micr.70109)
Supplement: Supplementary file 1 — Data S1: Supporting Information. [file MICR-45-e70109-s001.docx]

| **Study** | **D1** | **D2** | **D3** | **D4** | **D5** | **Overall Risk of Bias** |
| --- | --- | --- | --- | --- | --- | --- |
| Boccardo 2011 | Moderate | Low | Low | Low | Low | Moderate |
| Coriddi 2023 | Low | Low | Low | Low | Low | Low |

Supplementary Table S1. RoB 2.0 tool to assess risk of bias in randomized controlled studies, where D1 is risk of bias arising from the randomization process, D2 is risk of bias due to deviations from the intended interventions, D3 is risk of bias due to missing outcome data, D4 is risk of bias in measurement of the outcome, D5 is risk of bias in selection of the reported result (Sterne JAC, Savović J, Page MJ, Elbers RG, Blencowe NS, Boutron I, Cates CJ, Cheng H-Y, Corbett MS, Eldridge SM, Hernán MA, Hopewell S, Hróbjartsson A, Junqueira DR, Jüni P, Kirkham JJ, Lasserson T, Li T, McAleenan A, Reeves BC, Shepperd S, Shrier I, Stewart LA, Tilling K, White IR, Whiting PF, Higgins JPT. RoB 2: a revised tool for assessing risk of bias in randomised trials. *BMJ* 2019; **366**: l4898.)

| **Study** | **D1** | **D2** | **D3** | **D4** | **D5** | **D6** | **D7** | **Overall Risk of Bias** |
| --- | --- | --- | --- | --- | --- | --- | --- | --- |
| Haravu 2024 | Serious | Low | Low | Moderate | Moderate | Low | Low | Moderate |
| Le 2024 | Moderate | Low | Low | Moderate | Moderate | Low | Low | Moderate |
| Ozmen 2022 | Moderate | Low | Low | Moderate | Serious | Low | Low | Moderate |
| Herremans 2021 | Low | Low | Low | Moderate | Serious | Low | Low | Moderate |
| Chung 2023 | Low | Moderate | Low | Moderate | Serious | Low | Low | Moderate |
| Levy 2023 | Moderate | Low | Low | Low | Low | Low | Low | Low |
| Schwarz 2019 | Low | Low | Low | Low | Low | Low | Low | Low |
| Boccardo 2014 | Low | Low | Low | Low | Low | Low | Low | Low |
| Shaffer 2020 | Moderate | Low | Low | Moderate | Low | Low | Low | Moderate |
| Spoer 2024 | Low | Moderate | Low | Moderate | Moderate | Low | Low | Moderate |
| Granoff 2023 | Low | Low | Low | Moderate | Low | Low | Low | Low |
| Cook 2021 | Low | Low | Low | Moderate | Low | Low | Low | Low |
| Wainwright 2024 | Moderate | Moderate | Low | Moderate | Low | Low | Low | Moderate |
| Brahma 2024 | Moderate | Moderate | Low | Moderate | Moderate | Low | Low | Moderate |
| Wong 2024 | Moderate | Low | Low | Low | Low | Low | Low | Low |

Supplementary Table S2. ROBINS-I tool to assess risk of bias in non-randomized interventions, where D1 is bias due to confounding, D2 is bias in selection of participants into the study, D3 is bias in classification of interventions, D4 is bias due to deviation from intended interventions, D5 is bias due to missing data, D6 is bias in measurement of outcomes, and D7 is bias in selection of the reported result (Sterne JAC, Hernán MA, Reeves BC, Savović J, Berkman ND, Viswanathan M, Henry D, Altman DG, Ansari MT, Boutron I, Carpenter JR, Chan AW, Churchill R, Deeks JJ, Hróbjartsson A, Kirkham J, Jüni P, Loke YK, Pigott TD, Ramsay CR, Regidor D, Rothstein HR, Sandhu L, Santaguida PL, Schünemann HJ, Shea B, Shrier I, Tugwell P, Turner L, Valentine JC, Waddington H, Waters E, Wells GA, Whiting PF, Higgins JPT. ROBINS-I: a tool for assessing risk of bias in non-randomized studies of interventions. BMJ 2016; 355; i4919; doi: 10.1136/bmj.i4919.)
